# Formulation Matters: Differential Genotoxic and Cytotoxic Effects of Lambda-Cyhalothrin Pesticide Formulations on Human Hepatocellular Cells

**DOI:** 10.3390/jox16030098

**Published:** 2026-05-31

**Authors:** Khadija Ramadhan Makame, Moustafa Sherif, Le Vinh Hoi Thong, Balázs Ádám, Károly Nagy

**Affiliations:** 1 Department of Public Health and Epidemiology, Faculty of Medicine, University of Debrecen, 4032 Debrecen, Hungary; khadija.makame@med.unideb.hu (K.R.M.); tlevinh@mailbox.unideb.hu (L.V.H.T.); 2 Doctoral School of Health Sciences, University of Debrecen, 4032 Debrecen, Hungary; 3 Institute of Public Health, College of Medicine and Health Sciences, United Arab Emirates University, Al Ain 15551, United Arab Emirates; badam@uaeu.ac.ae; 4 Department of Anatomy and Embryology, Benha University, Benha City 13511, Egypt; mustafa.shref@fvtm.bu.edu.eg

**Keywords:** lambda-cyhalothrin, pesticide formulations, cytotoxicity, genotoxicity, micronucleus assay, comet assay, HepG2 cells

## Abstract

Pesticide formulations may influence toxicological outcomes beyond the intrinsic properties of active ingredients; however, these differences are often overlooked in regulatory risk assessment. Using human liver HepG2 cells, this study compared the cytotoxic and genotoxic effects of two commercial lambda-cyhalothrin formulations that differ in formulation type and in composition: an emulsifiable concentrate (Lambda-Cyhalothrin 5% EC) and a suspension concentrate co-formulated with thiamethoxam (Duer SC). Cytotoxicity was assessed using propidium iodide exclusion, while genotoxicity was evaluated using the cytokinesis-block micronucleus (CBMN) and alkaline comet assays. Lambda-Cyhalothrin 5% EC showed significant cytotoxicity from 500 μM onward, whereas Duer SC induced cytotoxic effects even at lower concentrations beginning at 200 μM, indicating greater cytotoxic potency of the combined formulation. Lambda-Cyhalothrin 5% EC induced a concentration-dependent increase in the number of binucleated cells containing micronuclei, with a significant effect at 50 μM. Duer SC also increased micronucleus frequency but did not differ significantly from the negative control. Proliferation indices remained comparable to controls for both formulations. The comet assay showed that Lambda-Cyhalothrin 5% EC produced significant DNA damage from 10 μM onward, while Duer SC exhibited minimal genotoxic effects. These findings demonstrate that formulation type modifies the toxicological profile of lambda-cyhalothrin.

## 1. Introduction

The use of pesticides in agriculture has grown into a global phenomenon with potential long-term health implications beyond their intended pest control applications. While regulatory agencies typically assess acute toxicity before market approval, chronic low-level exposures are increasingly recognized as significant public health concerns [1]. Of particular concern are the genotoxic, carcinogenic, and reproductive effects that certain pesticides may induce through prolonged exposure [2,3].

The genotoxic properties of various pesticides have been documented through multiple in vitro and in vivo studies, demonstrating their ability to damage genetic material in living cells [4,5,6,7]. This damage frequently results from oxidative stress mechanisms, with pesticides generating reactive oxygen species that subsequently compromise DNA integrity [3,8,9]. The World Health Organization has consequently urged governments to develop tools for selecting less dangerous pesticides and ensuring their appropriate application [10].

Regulatory frameworks for pesticides, including those established by the US Environmental Protection Agency (EPA) and the European Union’s Directive 91/414/EEC, primarily focus on evaluating the adverse effects of active ingredients and select representative formulations [11,12]. However, these assessments often overlook potential interactions between active ingredients and other components in commercial formulations, highlighting significant gaps in current regulatory approaches [13].

Formulation type significantly influences not just how pesticides are delivered but also their toxicological profiles and behavior in biological systems. Emulsifiable concentrates (ECs) contain active ingredients dissolved in petroleum-based solvents with emulsifiers to facilitate water mixing, creating an emulsion that enhances penetration into target tissues. In EC formulations, the dissolved active ingredient may increase bioavailability and cellular uptake compared to other formulations. In contrast, suspension concentrates (SCs) contain solid particles of active ingredients suspended in a liquid medium, allowing for potentially different absorption and interaction patterns with biological surfaces. Research has shown that these fundamental differences in formulation can lead to order-of-magnitude differences in pesticide behavior, with EC formulations often demonstrating enhanced penetration and bioavailability while SC formulations may exhibit different retention and dissipation dynamics [14,15,16]. These formulation-dependent properties extend beyond mere physical differences to potentially affect toxicological outcomes, including cytotoxicity and genotoxicity profiles, in ways not predictable from active ingredient assessment alone. Of greater concern is that different formulation types (such as emulsifiable concentrates versus suspension concentrates) are rarely compared directly in toxicological evaluations, despite evidence suggesting substantial differences in their safety profiles.

Pyrethroids, such as lambda-cyhalothrin (LCT), constitute a relatively recent class of insecticides characterized by rapid environmental degradation and reduced toxicity to mammals compared to traditional pesticide classes like organophosphates and organochlorines. Approved by the US EPA in 1988, LCT exerts its insecticidal effects by interfering with voltage-gated sodium channels in the insect nervous system, disrupting neural signalling and ultimately causing paralysis and death [17]. Despite its widespread use, the genotoxic potential of LCT remains controversial, with conflicting research findings. Some studies report significant DNA damage and mutations [18,19,20], while others suggest relative safety [21,22]. Research by Fetoui et al. (2015) [9] identified increased micronuclei frequency and oxidative stress markers in rats exposed to LCT, suggesting potential genotoxic mechanisms.

The increasing trend toward combination products containing multiple active ingredients, such as lambda-cyhalothrin with neonicotinoids like thiamethoxam, presents additional complexity for toxicological assessment. These combinations may produce interactive effects that either enhance or mitigate the toxicity of individual components [23]. Understanding how such combinations in different formulation types affect genotoxicity is critical for comprehensive risk assessment, particularly as manufacturers continue to develop new multi-active products. Notably, formulation-dependent toxicity remains understudied despite evidence that “inert” ingredients and formulation methods may significantly modify toxicological outcomes. This reveals a critical gap in current pesticide safety assessment, as regulatory evaluations generally prioritize active ingredients while overlooking the toxicity of full commercial formulations [24].

Two complementary genotoxicity assays, i.e., the cytokinesis-block micronucleus (CBMN) assay and comet assay, were used in this study to provide an integrated evaluation of DNA and chromosomal damage. The cytokinesis-block micronucleus (CBMN) assay is a widely applied cytogenetic method for detecting chromosomal damage and genome instability [25]. It enables the identification of both clastogenic events, resulting from chromosome breakage, and aneugenic effects arising from whole chromosome loss during cell division. Standardized under OECD Test Guideline 487, the CBMN assay is considered a reliable and sensitive approach for assessing chromosomal damage in human peripheral blood lymphocytes. Due to its ability to detect accumulated genetic damage following exposure, the micronucleus test is extensively used in genotoxicity assessments of environmental chemicals and pesticide formulations [26].

In addition to the CBMN assay, the comet assay (OECD TG 489) represents a validated and highly sensitive method for detecting DNA damage at the cellular level [26,27]. It allows the detection of primary DNA lesions, including single- and double-strand breaks and alkali-labile sites. While the CBMN assay reflects fixed chromosomal alterations, the comet assay identifies early and potentially reversible DNA strand damage. The recognition of both methods as OECD test guidelines underscores their regulatory relevance. When used in combination, these assays offer a layered and thorough assessment of genotoxicity by covering both initial DNA damage and chromosomal instability. This makes them especially well-suited for evaluating the genotoxic impact of pesticide formulations [28,29,30,31]. This study aimed to compare the cytotoxic and genotoxic potential of lambda-cyhalothrin in two commercially available pesticide formulations: an emulsifiable concentrate (Lambda-Cyhalothrin 5% EC) and a suspension concentrate containing an additional active ingredient, thiamethoxam (Duer SC). The comparison therefore involved formulations that differed both in formulation type (EC versus SC) and in composition (single versus dual active ingredient). Consequently, any observed toxicological differences may reflect the independent or interactive effects of formulation characteristics, co-formulants, and active ingredient composition. Within this framework, the study was designed to determine whether the two commercial formulations differ in the concentration ranges at which cytotoxic and genotoxic effects are induced in human hepatocellular (HepG2) cells, and to evaluate whether the presence of thiamethoxam modifies the toxicological profile associated with lambda-cyhalothrin relative to the single-active-ingredient EC formulation.

## 2. Materials and Methods

### 2.1. Pesticide Formulations and Preparation

Two lambda-cyhalothrin formulations were evaluated in this study. Lambda-Cyhalothrin 5% EC was obtained from Agri Sciences Ltd., Izmir, Turkey (MOCCAE registration: DXB-AD-140-1507847), containing 5% lambda-cyhalothrin and 95% inert ingredients (molecular weight: 449.85 g/mol; relative density: 1.33 g/cm^3^). PAN 247 g/L SC (Duer SC) was procured from Shandong United Pesticide Industry, Tai’an, China (MOCCAE registration: AUD-AD-140-2343936), containing 141 g/L thiamethoxam, 106 g/L lambda-cyhalothrin, and 753 g/L inert components. For the Lambda-Cyhalothrin 5% EC formulation, a 10,000 μM stock solution was prepared by diluting 135.293 μL of the pesticide in 1864.707 μL of culture medium. For the PAN 247 g/L SC formulation, 84.877 μL of the pesticide was diluted in 1915.123 μL of culture medium to achieve a 10,000 μM stock solution. Serial dilutions of both formulations were prepared in RPMI medium to obtain final concentrations of 0.01, 0.1, 1, 10, 50, 100, 200, 500, and 1000 μM for the cytotoxicity and comet assays. For the cytokinesis-block micronucleus (CBMN) assay, serial dilutions were prepared separately in DMEM to achieve final concentrations of 0.1, 1, 10, 50, 100, and 200 μM.

### 2.2. Cell Culture

Human hepatocellular carcinoma cells (HepG2) were obtained from Prof. Bassam Ali’s laboratory at the College of Medicine and Health Sciences, United Arab Emirates University, who had previously purchased them from the ATCC (ATCC HB-8065), and had also been used in a previous study [32]. The adherent cell line was tested negative for mycoplasma at acquisition, and maintained in RPMI 1640 medium (Gibco, Grand island, NY, USA, Cat#: 11875093) supplemented with 10% fetal bovine serum (Gibco, Cat#: A5256701) and 1% Penicillin–Streptomycin (10,000 U/mL) (Gibco, Grand island, NY, USA, Cat#: 15140122) for cytotoxicity test and comet assay, and in Dulbecco’s Modified Eagle Medium (DMEM) (VWR International, Radnor, PA, USA, Cat#: vwr-MS02MY1) for CBMN assay. Cells were cultured under standard conditions and used for experiments when they reached approximately 80% confluence, with fresh medium provided 24 h before the experiments.

### 2.3. Cell Treatment

For cytotoxicity, HepG2 cells were exposed to increasing concentrations of 0.01, 0.1, 1, 10, 50, 100, 200, 500 and 1000 μM of the test chemicals in cell culture medium, with concentration selection guided by previous in vitro cytotoxicity studies of lambda-cyhalothrin demonstrating dose-dependent reductions in cell viability, with reported LC_50_/EC_50_ values generally occurring between approximately 28 and 75 µM, depending on the cell model and exposure duration [19,33,34]. The selected concentration range therefore encompassed sub-cytotoxic, moderately cytotoxic, and overtly cytotoxic doses to support appropriate range-finding. For genotoxicity, cells were exposed to 0.1, 1, 10, 50, 100 and 200 μM based on the results from the cytotoxicity assay, which indicated that cell viability started to deteriorate significantly from 200 μM. 100 μg/mL bleomycin was used as a positive control and DMEM as a negative control for the CBMN assay, while hydrogen peroxide (100 μM) served as a positive control and PBS was used as a negative control for the comet assay. All treatment was set up so as to keep the final volume concentration of the tested chemicals in the medium below 10% (*v*/*v*). The formulations were diluted directly in the cell culture medium. In the case of the 5% EC lambda-cyhalothrin formulation, the petroleum-based solvent carrier was co-diluted with the active ingredient at each concentration level, thereby maintaining the original formulation matrix during exposure. Cells were treated for 1 h at 37 °C under standard culture conditions before subsequent analyses. For the alkaline comet assay, HepG2 cells were exposed to the test formulations for 1 h prior to slide preparation. This short-term exposure design was selected in accordance with established comet assay protocols and the recommendations of OECD Test Guideline 489, which recognize exposure periods of approximately 1–2 h as suitable for the detection of primary DNA damage, including single-strand breaks and alkali-labile sites, before the onset of extensive DNA repair processes or secondary cytotoxic effects. Consequently, the short exposure duration enables the assessment of early genotoxic events while minimizing confounding influences associated with prolonged cellular stress and repair-related alterations in DNA integrity. In contrast, for the cytokinesis-block micronucleus (CBMN) assay, cells were exposed to the test formulations for 20 h prior to the addition of cytochalasin B, in accordance with OECD Test Guideline 487 recommendations. This treatment duration approximately corresponds to one cell cycle in proliferating mammalian cells and is considered appropriate for allowing chromosome damage or mitotic dysfunction to be expressed as micronuclei in binucleated cells.

### 2.4. Cytotoxicity Assessment

Cell viability was assessed immediately following treatment using propidium iodide (PI) fluorescent dye, which permeates the membranes of dead and dying cells but is excluded from viable cells with intact membranes. PI was dissolved in PBS (pH 7.2) to achieve a final concentration of 2 μM. Cell pellets containing 1 × 10^5^ cells were incubated with 200 μL of the PI working solution for 30 min at 4 °C, protected from light. After labeling, cells were washed and resuspended in ice-cold PBS buffer. For microscopic examination, 40 μL of cell suspension was placed on a microscope slide and immediately examined under a Zeiss epifluorescent microscope at 20× magnification. Total cell counts were determined using transmitted light microscopy on two non-overlapping fields per slide, while PI-labeled cells were visualized using a TRITC filter. Cell viability was calculated by subtracting the number of PI-fluorescent (non-viable) cells from the total cell count and expressing this value as a proportion of the total cells counted. For each experiment, cell viability (%) was calculated using the following formula:

$$Cell\;viability\left(\%\right)=\left(total\;number\;of\;cells-number\;of\;PI\;fluorescent\;cells|total\;number\;of\;cells\right)\times 100$$

The mean cell viability from repeated independent experiments was used to represent overall cell viability.

### 2.5. Genotoxicity Assessment

#### 2.5.1. Cytokinesis-Block Micronucleus Assay

The cytokinesis-block micronucleus (CBMN) assay was performed, in accordance with the OECD Test Guideline 487 for the In Vitro Mammalian Cell Micronucleus Test [25], and the protocol described by Bolognesi and Fenech [35], with minor laboratory-specific modifications. HepG2 cells were cultured in Dulbecco’s Modified Eagle Medium (DMEM) supplemented with 10% fetal bovine serum (FBS), 2% L-glutamine, 1% penicillin, 1% streptomycin, and 1.8% Phytohemagglutinin (PHA) (Biosera; Cholet, France). For each experimental condition, 2–2.5 × 10^5^ cells/well were seeded in a 6-well plate and incubated at 37 °C with 5% CO_2_ for 24 h prior to chemical exposure. After the 24 h pre-incubation, cell cultures were exposed to lambda-cyhalothrin or Duer SC at the specified concentrations. For each treatment, 250 μL of the prepared working solution was added to 2250 μL of culture medium to achieve the target exposure concentration. Cells were exposed for 20 h under standard incubation conditions. Following exposure, the medium was aspirated, cells were washed with HBBS, and 2471 μL of fresh medium was added to each well. Cytokinesis was inhibited using Cytochalasin-B (3.5 μg/mL). A volume of 29 μL from a 300 μg/mL Cyt-B stock solution was added to each culture (final volume 2.5 mL). Cultures were incubated for 28 h. After 28 h, the medium was aspirated, cells were rinsed with HBBS, and 0.5 mL trypsin was added. After incubation (5–7 min at 37 °C), detached cells were transferred into centrifuge tubes containing 3 mL of complete medium. Samples were centrifuged at 1000 rpm for 5 min at 4 °C; 50–100 μL of residual volume was retained. Cells were gently mixed with 0.5 mL of 0.075 M KCl to induce mild hypotonic swelling. Cells were fixed by adding 5 mL cold methanol: acetic acid (3:1) and incubated for 10 min on ice. The fixation step was repeated two additional times after centrifugation (1000 rpm, 5 min). The final pellet was resuspended in ~200 μL of fixing solution. Microscope slides were cleaned with 70% ethanol and air-dried. A few drops (1–2 per slide) of the resuspended cell suspension were dropped from a height of 10–20 cm to produce optimally spread nuclei. Slides were dried at room temperature, then stained using 3% Giemsa solution prepared with Sorensen buffer (pH 6.8). The slides were subsequently rinsed in Sorensen buffer, thoroughly dried, and mounted utilizing Eukitt^®^ mounting medium (Sigma-Aldrich Co., St. Louis, MO, USA).

Micronucleus scoring was performed according to OECD TG 487 criteria [25]. For each treatment condition, 1000 binucleated cells were examined at 400× magnification using a Zeiss Axiocam 503 mono digital camera (Carl Zeiss AG, Oberkochen, Germany). Slide scoring was performed by two independent evaluators; each experiment included two replicate slides. The proliferation index (PI) was calculated by analyzing at least 1000 cells categorized as mononucleated, binucleated, or multinucleated, using the following formula:

$$PI\;=\;\left[\left(nMONO\right)+2\left(nBN\right)+3\left(nMULTI\right)\right]÷N$$

where

- ***PI*** = proliferation index;
- ***nMONO*** = number of mononucleated cells;
- ***nBN*** = number of binucleated cells;
- ***nMULTI*** = number of multinucleated cells;
- ***N*** = total number of cells evaluated.

The frequency of micronuclei (BNMN%) in binucleated cells was obtained by dividing the number of binucleated cells containing micronuclei by the total number of binucleated cells.

#### 2.5.2. Comet Assay

DNA damage was evaluated using the alkaline comet assay as described by Collins (2004) [30]. Following treatment, HepG2 cells were centrifuged and resuspended in PBS at a density of 1800 cells/μL. Frosted degreased slides were prepared with a base layer of 1% normal melting point agarose (NMA) followed by a layer of 0.75% low melting point agarose (LMA) containing the cell suspension (2 × 10^5^ cells per slide). After 10 min of solidification at 4 °C, embedded cells underwent overnight lysis using Comet Assay Lysis Solution (R&D Systems, Minneapolis, MN, USA, Cat#4250-050-01) at 4 °C, protected from light. The DNA was then allowed to unwind for 20 min in alkaline unwinding solution (300 mM NaOH, 1 mM EDTA) before electrophoresis in the same solution for 20 min at 1 V/cm and 300 mA. Slides were subsequently rinsed with Milli-Q water to remove residual alkali and detergent, washed twice with 70% ethanol, and allowed to dry for 10 min in darkness. Each slide was stained with PI (20 μg/mL), washed with water, covered with a coverslip, and stored in a humidified container until analysis.

Fluorescence signals were captured at 40× magnification using the Metafer automated image analysis system (MetaSystems GmbH, Altlussheim, Germany) equipped with the ZEISS Axio Imager Z2 and comet can module. The Neon Software (Neon v1.3) was used to analyse more than 100 randomly captured comet images from duplicate slides per concentration and to compute DNA damage parameters. Tail DNA percentage, tail length (μm), tail moment, and Olive tail moment were quantified to comprehensively assess DNA damage. Results were presented as the mean of the median values of DNA damage parameters from two independent experiments.

### 2.6. Data Analysis

**Cell 100.** minus cytotoxicity, represented by the mean proportion of living cells from three repeated experiments. Arithmetic mean cell viability values at each tested concentration were compared with the untreated negative control using one-way ANOVA followed by Dunnett’s post hoc test. In addition, to enable direct quantitative comparison of concentration–response relationships between formulations, a slope assay was conducted in accordance with the approach recommended for proportion data. Cell viability proportions were logit-transformed as *l = ln[(p + 0.005)/(1 − p + 0.005)]*, where *p* = viability/100, and regressed against log_10_ concentration using a linear model. The slope of this regression (viability on log_10_ concentration) and its standard error were estimated for each formulation, and a *t*-test was used to assess whether each slope was significantly negative, indicating concentration-dependent toxicity. Concentration values were plotted on a log_10_ scale. The full results including mean differences, standard errors, and exact *p*-values are provided in the Supplementary Statistical Table S2. For the CBMN assay, results are presented as means with standard errors of the mean (SEM), obtained from three independent experiments, each carried out in duplicate. One-way analysis of variance (ANOVA), followed by Dunnett’s post hoc test, was used to assess differences in frequency of micronuclei (BNMN%) and proliferation index (PI) between treated samples and untreated controls. For comet analysis, only slides with more than 100 images per concentration for each experiment were included in the analysis. The median values of comet assay metrics (percentage of DNA in the tail, tail length, tail moment, and Olive tail moment) at various concentrations were calculated, and the arithmetic means of the medians from two independent experiments were determined. The mean of median comet assay metrics across independent experiments at each concentration was compared to those of the untreated negative control using one-way ANOVA followed by Dunnett’s post hoc test. Error bars in figures represent ±1 standard error (SE) to illustrate the variability and reliability of the data across different experiments. Full results including mean differences, standard errors, and exact *p*-values are provided in the Supplementary Statistical Table S3. IBM SPSS Statistics (Version 29.0) was used for statistical analysis of frequency of micronuclei and proliferation index, whereas all other statistical analyses and figure generation were performed using R (version 4.3 or later; R Core Team, 2024) in RStudio (version 2024.04 or later; Posit, PBC). Statistical significance was accepted at 5% level (*p* < 0.05).

## 3. Results

### 3.1. Cytotoxicity

**Figure 1:**
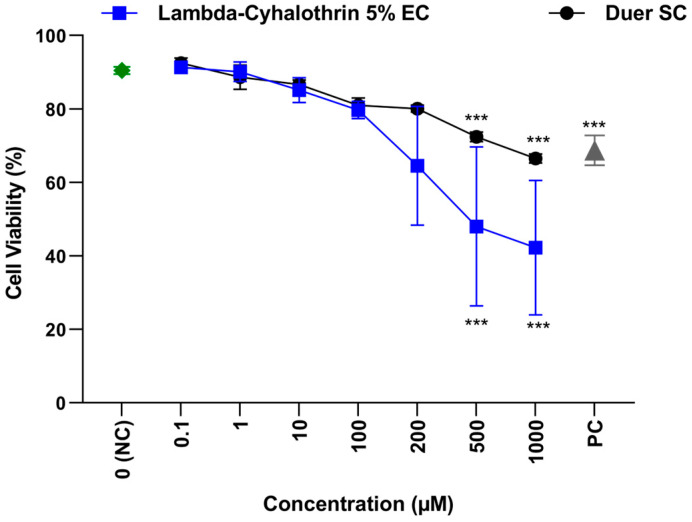
Effect of Lambda-Cyhalothrin 5% EC and Duer SC formulations on the viability of HepG2 cells. Cells were exposed to increasing concentrations of the formulations. NC (green cube, negative control); PC (gray triangle, positive control). Data represent mean ± SEM from three independent experiments. Statistical significance compared to the negative control was determined by one-way ANOVA followed by Dunnett’s post hoc test (*** *p* ≤ 0.001).

Cytotoxicity demonstrated a concentration-dependent decrease in cell viability for both Lambda-Cyhalothrin 5% EC and Duer SC formulations (Figure 1). At lower concentrations (0–1 μM), both formulations maintained high cell viability (>90%). As concentrations increased, a progressive decline in cell viability was observed, with statistical significance occurring at different concentration thresholds for each formulation. Lambda-Cyhalothrin 5% EC demonstrated significant cytotoxicity beginning at 500 µM (mean difference: −20.72 ± 3.65%, *p* = 0.001), which further increased at 1000 µM (*p* < 0.001). In comparison, Duer SC exhibited significant cytotoxicity at a lower concentration of 200 µM (mean difference: −10.47 ± 2.77%, *p* = 0.020), with more pronounced effects at 500 µM (*p* < 0.001) and 1000 µM (*p* < 0.001); full Dunnett’s post hoc results are presented in Supplementary Table S2. To quantify and directly compare the concentration–response relationships between the two formulations, a slope assay was conducted by logit-transforming viability proportions and regressing these against log_10_ concentration. Both formulations showed a statistically significant negative slope, confirming concentration-dependent cytotoxicity: Lambda-Cyhalothrin 5% EC (slope = −0.37 ± 0.07, *p* < 0.001) and Duer SC (slope = −0.34 ± 0.05, *p* < 0.001). The slopes were comparable in magnitude, indicating similar rates of viability decline per log-unit increase in concentration for both formulations (Supplementary Table S1).

### 3.2. Micronucleus Formation

**Figure 2:**
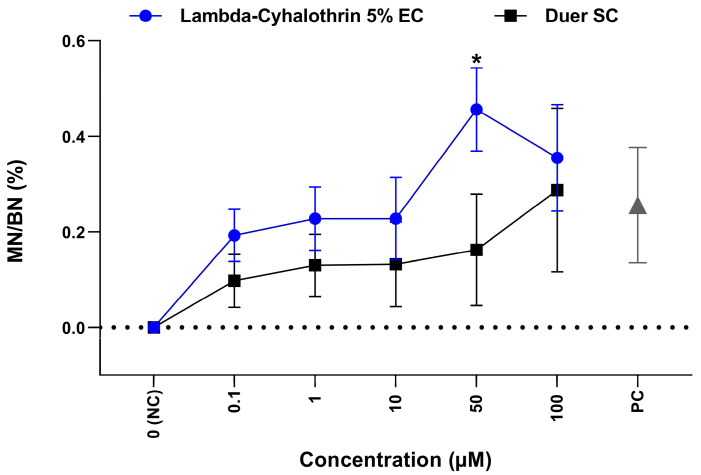
Effect of Lambda-Cyhalothrin 5% EC and Duer SC formulations on micronucleus formation in HepG2 cells. Frequency of binucleated cells with micronuclei (BNMN%) was evaluated using the cytokinesis-block micronucleus assay. NC (negative control), PC (gray triangle, positive control). Data represent mean ± SEM from three independent experiments. Statistical significance compared to the untreated control was determined by one-way ANOVA followed by Dunnett’s post hoc test (* *p* < 0.05).

Based on the cytotoxicity results, genotoxicity testing was conducted at concentrations maintaining ≥80% cell viability to avoid confounding effects. Both the Lambda-Cyhalothrin 5% EC and Duer SC formulation induced increases in total micronuclei frequency compared with the negative control (Figure 2). Lambda-Cyhalothrin 5% showed a more pronounced genotoxic response, particularly at intermediate concentrations, whereas Duer SC formulation elicited a moderate but consistent increase across concentrations. A statistically significant increase was observed only with Lambda-Cyhalothrin 5% EC at 50 µM (*p* = 0.019). The positive control demonstrated elevated MN levels, while the negative control showed minimal background damage. The proliferation index remained relatively stable across all tested concentrations of Lambda-Cyhalothrin 5% EC and Duer SC formulation, with values comparable to the negative control. No marked concentration-dependent reduction in PI was observed. The positive control showed a slight reduction in PI (Table 1).

### 3.3. DNA Damage

**Figure 3:**
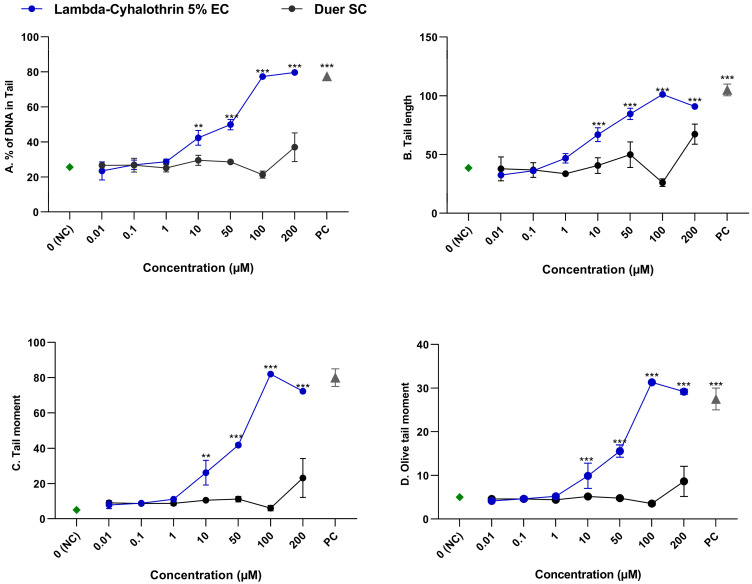
Genotoxicity effects of Lambda-Cyhalothrin 5% EC and Duer SC formulations in HepG2 cells assessed by the comet assay. A: DNA tail percentage; B: tail length; C: tail moment; D: Olive tail moment. NC (green cube-negative control), PC (gray triangle-positive control). Data represent mean ± SEM from two independent experiments. Statistical significance compared to the negative control (NC) was determined by one-way ANOVA followed by Dunnett’s post hoc test ( ** *p* < 0.01, *** *p* < 0.001).

The comet assay revealed substantial differences between the two formulations across all measured DNA damage parameters (Figure 3). Lambda-Cyhalothrin 5% EC consistently induced significant concentration-dependent increases in all genotoxicity markers beginning at 10 μM. Specifically, at this lowest effective concentration, significant increases were observed in DNA tail percentage (mean difference: 18.53 ± 5.01%, *p* = 0.009), tail length (mean difference: 28.98 ± 8.78 µm, *p* = 0.023), tail moment (mean difference: 16.552 ± 4.48, *p* = 0.009), and Olive tail moment (mean difference: 5.10 ± 1.50, *p* = 0.018). Full results for all concentrations and both formulations are presented in Supplementary Table S3. All four parameters showed progressively larger effects at higher concentrations (50 μM, 100 μM, and 200 μM; all *p* < 0.001), demonstrating a clear dose–response relationship.

In contrast, Duer SC exhibited minimal genotoxic potential across the tested concentration range. Among the four parameters measured, only tail length showed a statistically significant increase, and this occurred only at the highest tested concentration of 200 μM (mean difference: 29.471 ± 9.863, *p* = 0.039). No significant changes were detected for DNA tail percentage, tail moment, or Olive tail moment at any tested concentrations, with values remaining comparable to negative control levels throughout the concentration range. Only tail length showed a statistically significant increase at 200 µM (mean difference: +29.47 ± 9.68 µm, *p* = 0.040), coinciding with the onset of significant cytotoxicity at this concentration. It should be noted that for tail length, tail moment, and Olive tail moment, variance increased at higher concentrations, consistent with the known distributional properties of comet assay data and significance levels for these parameters should therefore be regarded as approximate (Supplementary Table S3).

## 4. Discussion

The present study provides compelling evidence that pesticide formulation type significantly influences the genotoxic and cytotoxic profile of lambda-cyhalothrin in human hepatocellular cells. Our findings demonstrate substantial differences between the emulsifiable concentrate (Lambda-Cyhalothrin 5% EC) and suspension concentrate (Duer SC) formulations, highlighting the critical importance of considering complete formulations rather than solely active ingredients when evaluating pesticide safety.

Lambda-Cyhalothrin 5% EC demonstrated clear genotoxic potential at concentrations that did not produce marked cytotoxicity. Micronucleus analysis revealed a concentration-dependent increase in micronucleated binucleated cells (BNMN%), with a statistically significant elevation at 50 µM and more pronounced responses at 50–100 µM. Although Duer SC also increased BNMN% frequency, the magnitude of induction was consistently lower than that of the emulsifiable concentrate, indicating formulation-dependent differences in genotoxic potency. Importantly, the MN induction observed at 50 µM occurred under conditions of preserved cell viability (approximately ≥80% in HepG2 cells), while pronounced cytotoxicity was detected only at substantially higher concentrations (≥200–500 µM). This dissociation between genotoxicity and cytotoxicity is critical for interpretation, as OECD TG 487 emphasizes evaluating micronucleus formation at concentrations that do not markedly inhibit cell division or induce excessive cytotoxicity, a principle broadly applicable across genotoxicity assays. Consistent with the micronucleus findings, comet assay results showed significant DNA damage at an even lower concentration (10 µM), further supporting the presence of a primary genotoxic effect rather than secondary damage arising from nonspecific cellular toxicity. At higher cytotoxic concentrations, genotoxic endpoints may become confounded by severe cellular stress [36], highlighting the 10–50 µM range as particularly relevant for genotoxic hazard identification.

Collectively, in vitro evidence demonstrates that lambda-cyhalothrin (LCT) exerts pronounced cytotoxic effects across multiple cell systems, frequently accompanied by DNA damage, with dose, exposure duration, and cellular context acting as critical modifiers. In human peripheral blood lymphocytes, LCT induced clear dose-dependent cytotoxicity, as reflected by a progressive decline in cell viability and an LC_50_ of 28 µM, suggesting relatively high cytotoxic potency among pyrethroids [33]. Meanwhile, comet assay findings revealed a significant, dose-related increase in DNA single-strand breaks, with elevated tail lengths detected even at sub-cytotoxic concentrations (1/10 of the LC_50_; *p* < 0.02), indicating that genotoxic effects may precede overt cytotoxicity [33].

Time-dependent toxicity has also been demonstrated in human intestinal Caco-2 cells following repeated LCT exposure, where cumulative cytotoxicity became evident over prolonged exposure periods. The concentration required to produce 50% of the maximal biological effect (EC_50_) declined progressively, reaching approximately 60 µM after 10 days, indicating bioaccumulative or delayed cellular injury. At the cellular level, this toxicity was closely associated with oxidative stress, as evidenced by increased hydrogen peroxide production, lipid peroxidation, and disrupted antioxidant enzyme activities (CAT, GPx, and SOD). The partial mitigation of cytotoxicity by antioxidant pretreatment further supports a pro-oxidative mode of action underlying LCT-induced cellular damage [34].

Genotoxic effects of LCT have been consistently reported in non-human cell models as well. In Sf9 insect cells, alkaline comet assay analysis demonstrated significant DNA damage following 24 h exposure, with damage levels increasing across concentrations from 0.5 to 100 µM and peaking at the highest dose [19]. Similarly, Laborde et al. (2023) reported a dose-dependent induction of DNA single-strand breaks, characterized by increased proportions of highly damaged (type III–IV) comets at 10 and 100 µg/mL [37]. Notably, while DNA migration increased, the overall genetic damage index was less pronounced for the active ingredient than for the formulated product, highlighting the potential contribution of co-formulants to genotoxic outcomes.

Evidence from cytokinesis-block micronucleus (CBMN) assays further underscores the dominant cytotoxic profile of LCT in human lymphocytes. Muranli (2013) [38] reported marked cytotoxicity across all tested concentrations (1–30 µM), evidenced by a significant reduction in the Nuclear Division Index (*p* < 0.001), with complete inhibition of cell division at concentrations ≥3.75 µM. Although micronucleus induction was observed at lower concentrations (1–2 µM), genotoxic effects were largely obscured at higher doses due to severe cytostatic and suppression of binucleated cell formation [38]. This finding underscores the importance of carefully interpreting genotoxic endpoints under conditions of high cytotoxic stress.

Importantly, these in vitro observations are supported by human biomonitoring data, which provide biological plausibility for adverse cellular effects in exposed populations. Agricultural workers exposed to pesticides, including lambda-cyhalothrin, exhibited significantly elevated frequencies of cytotoxic and apoptotic nuclear abnormalities such as karyolysis, pyknosis, karyorrhexis, condensed chromatin, and binucleated cells in buccal epithelial cells. Moreover, a marked increase in micronucleate buccal cells was observed, with exposed farmers showing approximately a 20-fold higher median micronucleus frequency compared with non-farmers (714 vs. 36; *p* < 0.001), indicating substantial chromosomal damage and genomic instability [39].

Overall, the available evidence indicates that lambda-cyhalothrin induces robust cytotoxicity and measurable genotoxic effects in vitro, largely mediated by oxidative stress and exacerbated by prolonged exposure. The frequent masking of genotoxic endpoints by pronounced cytostatic highlights the need for careful dose selection and complementary mechanistic assays when evaluating the genotoxic potential of highly cytotoxic pesticides.

This separation between genotoxic and cytotoxic thresholds raises important considerations for risk assessment, as traditional cytotoxicity screenings might miss significant genotoxic effects occurring at lower, seemingly non-toxic concentrations. These findings align with previous research by Fetoui et al. (2015), who reported increased micronuclei frequency in rats exposed to lambda-cyhalothrin, suggesting consistent evidence for genotoxic potential across different experimental models [9].

In contrast, the Duer SC formulation demonstrated a notably different toxicological profile. Despite containing the same active ingredient (lambda-cyhalothrin), this suspension concentrate exhibited minimal genotoxic effects across most parameters, with only one comet assay parameter (tail length) showing significant effects at the highest tested concentration (200 μM). Interestingly, this concentration coincided with the threshold for significant cytotoxicity for this formulation. The substantial reduction in genotoxic potential observed with Duer SC suggests that formulation characteristics play a pivotal role in modulating the biological interactions of lambda-cyhalothrin.

Several factors may contribute to the observed differences between formulations. First, the physical state of lambda-cyhalothrin differs between formulations dissolved in petroleum-based solvents in the EC formulation versus solid particles suspended in a liquid medium in the SC formulation. This fundamental difference likely affects bioavailability and cellular uptake, with the dissolved state in EC formulations potentially enhancing penetration into cellular structures and increasing interactions with genetic material [14,15,16].

Second, the presence of thiamethoxam as a secondary active ingredient in Duer SC may provide protective effects against lambda-cyhalothrin-induced genotoxicity. Previous research has demonstrated that certain pesticide combinations can produce antagonistic effects on toxicity parameters [23]. The neonicotinoid thiamethoxam has a different mode of action from pyrethroids, targeting nicotinic acetylcholine receptors rather than sodium channels [40], which may influence cellular response pathways when combined with lambda-cyhalothrin.

Third, the “inert” components in each formulation, comprising 95% of Lambda-Cyhalothrin 5% EC and 753 g/L (75.3%) of Duer SC, may significantly modify the toxicological properties of the active ingredients. These components, often considered toxicologically irrelevant by regulatory frameworks, clearly play a substantial role in determining the ultimate safety profile of commercial formulations. The specific emulsifiers, solvents, and additives in each formulation likely influence cellular uptake, subcellular distribution, and interaction with biological structures [14,24,41].

The differential genotoxic responses observed between formulations may also reflect varying capacities to induce oxidative stress. Previous studies have established that many pesticides, including lambda-cyhalothrin, can induce DNA damage through the generation of reactive oxygen species [3,8,9,39,42]. The pronounced genotoxicity of Lambda-Cyhalothrin 5% EC in BNMN% as well as across all comet parameters may suggest a robust oxidative stress response, potentially exacerbated by petroleum-based solvents in the formulation [43]. Conversely, the minimal genotoxicity of Duer SC may indicate reduced oxidative stress induction, possibly due to different formulation components or interactions between lambda-cyhalothrin and thiamethoxam affecting redox balance in exposed cells [44].

Our findings highlight a critical gap in current regulatory approaches to pesticide safety assessment. The substantial differences observed between formulations containing the same active ingredient underscore the necessity for comprehensive evaluation of complete commercial formulations rather than relying primarily on active ingredient testing. This aligns with growing concerns in the scientific community regarding the limitations of current regulatory frameworks [13,45] and supports the World Health Organization’s call for developing tools to select less dangerous pesticides and ensure their appropriate application. For the pesticide industry, these results provide valuable insights for formulation development aimed at reducing genotoxic potential while maintaining efficacy. The potential protective effect of combining lambda-cyhalothrin with thiamethoxam merits further investigation as a strategy for developing safer multi-active ingredient formulations.

The present findings underscore significant regulatory challenges associated with lambda-cyhalothrin, particularly when formulated as nano- or micro-encapsulated products. Current pesticide regulatory frameworks primarily assess risks based on the active ingredient and conventional formulations, with limited consideration given to formulation-specific properties such as particle size, encapsulation matrix, and release kinetics. However, evidence demonstrates that nano-sized lambda-cyhalothrin formulations exhibit enhanced bioavailability, increased tissue accumulation, and higher acute toxicity to non-target aquatic organisms compared with micro-sized formulations and emulsifiable concentrates [46]. Although direct genotoxic endpoints are not always evaluated, formulation-driven increases in oxidative stress, cellular disruption, and environmental persistence suggest a plausible elevation of indirect genotoxic risk. However, occupational relevance can be inferred indirectly from published biomonitoring and human biomarker studies. In occupational biomonitoring investigations conducted among strawberry field workers, urinary concentrations of the lambda-cyhalothrin metabolite CFMP, together with 3-PBA and 4-OH3PBA, were quantified in serial urine samples, enabling characterization of exposure-related excretion kinetics [47]. In parallel, micronucleus cytome assays performed on buccal epithelial cells of agricultural workers occupationally exposed to pesticides demonstrated elevated micronucleus frequencies and other nuclear abnormalities, including in populations exposed to pyrethroid insecticides such as cypermethrin [44]. Although direct tissue-level concentrations in humans are not available, our in vitro findings obtained at 10–30 µM may therefore represent biologically plausible exposure conditions reflecting cumulative intracellular accumulation in chronically exposed hepatic tissue, particularly considering the liver’s central role in first-pass uptake. These mechanisms are insufficiently addressed in standard regulatory genotoxicity testing strategies, raising concerns that risks associated with nano-enabled pesticides may be underestimated. The observed formulation-dependent toxicity highlights the need for regulatory authorities to require nano-specific data, including particle size characterization, bioaccumulation studies, and chronic or sublethal toxicity endpoints. Given the recognized environmental risks of lambda-cyhalothrin and its regulatory restrictions in several jurisdictions, these findings support a precautionary, formulation-sensitive regulatory approach. Incorporating “safety-by-design” principles and differentiated risk assessments for nano-, micro-, and conventional formulations is essential to ensure effective pest control while minimizing cytotoxic and potential genotoxic risks to non-target organisms and ecosystems [48,49].

While this study provides important insights into formulation-dependent toxicity, several limitations should be acknowledged. First, the in vitro nature of our experiments, although appropriate for mechanistic investigations, may not fully recapitulate in vivo exposure scenarios. Although solvent-only control was not included, the potential impact of the vehicle is likely minimal, given the very low final solvent concentration, consistently low background damage in the untreated negative control, and the absence of comparable genotoxic effects in the SC formulation at equivalent volumes. Together, these observations suggest that the genotoxicity observed in the EC formulation is more likely attributable to its bioactive components rather than the solvent. Future studies should validate these findings in animal models with environmentally relevant exposure routes and durations.

Second, while we focused on DNA damage as detected by micronucleus formation and the comet assay, additional genotoxicity endpoints such as sister-chromatid exchange, chromosomal aberrations, and gene mutations would provide a more comprehensive assessment of genotoxic potential. Furthermore, investigating the molecular mechanisms underlying the observed formulation-dependent effects, particularly the role of oxidative stress markers and DNA repair pathways, would enhance our understanding of how formulation characteristics influence genotoxicity. Also, exploring a wider range of lambda-cyhalothrin formulations across different manufacturers would help determine whether the patterns observed in this study represent broader trends across the industry. Finally, investigating other pyrethroid insecticides in different formulation types would clarify whether our findings are specific to lambda-cyhalothrin or represent a more general phenomenon within this pesticide class.

It is important to note that the differences in toxicological responses observed between Lambda-Cyhalothrin 5% EC and Duer SC should not be interpreted as being attributable solely to the formulation type. The two commercial products differed both in formulation characteristics (EC versus SC) and in active ingredient composition, as Duer SC additionally contained thiamethoxam. Therefore, the observed differences in cytotoxicity and genotoxicity may result from independent or interactive effects of formulation properties, co-formulants, and active ingredient interactions. This distinction is important when interpreting the comparative toxicological profiles of the two products. Nevertheless, the present findings remain highly relevant from a regulatory and public health perspective because pesticide exposures in real-world settings occur through complete commercial formulations rather than isolated active ingredients. Our results therefore support the growing recognition that toxicological assessment should incorporate evaluation of marketed multi-component pesticide products, including potential interactions among formulation constituents and combined active ingredients, in order to more accurately characterize human exposure risks.

## 5. Conclusions

This study demonstrates that pesticide formulation type substantially influences the genotoxic and cytotoxic potential of lambda-cyhalothrin in human hepatocellular cells. The emulsifiable concentrate formulation (Lambda-Cyhalothrin 5% EC) induced significant DNA damage at concentrations far below cytotoxic thresholds, while the suspension concentrate formulation with thiamethoxam (Duer SC) exhibited minimal genotoxicity despite earlier onset of cytotoxicity. These findings emphasize the critical importance of evaluating a wide range of complete commercial formulations rather than focusing on active ingredients when assessing pesticide safety. Furthermore, this research aims to provide evidence-based insights for formulation development that may mitigate genotoxic potential while maintaining pesticide efficacy. By identifying the minimum concentration causing significant DNA damage and comparing different formulation types, this research will contribute to a more comprehensive understanding of how formulation techniques and combinations of active ingredients influence pesticide safety profiles. The findings may inform future regulatory approaches to evaluating commercial pesticide products beyond simple assessment of active ingredients.

## Figures and Tables

**Table 1:** Effect of Lambda-Cyhalothrin 5% EC and Duer SC formulations on proliferation index in HepG2 cells.

Formulation	Concentration (µM)	Mean ± SEM
Lambda-Cyhalothrin 5% EC	0.1	1.395 ± 0.023
Duer SC formulation		1.357 ± 0.016
Lambda-Cyhalothrin 5% EC	1	1.373 ± 0.022
Duer SC formulation		1.328 ± 0.031
Lambda-Cyhalothrin 5% EC	10	1.352 ± 0.020
Duer SC formulation		1.403 ± 0.016
Lambda-Cyhalothrin 5% EC	50	1.352 ± 0.010
Duer SC formulation		1.372 ± 0.023
Lambda-Cyhalothrin 5% EC	100	1.307 ± 0.012
Duer SC formulation		1.372 ± 0.010
Lambda-Cyhalothrin 5% EC	200	1.357 ± 0.022
Duer SC formulation		1.391 ± 0.024
Positive Control	100 μg/mL bleomycin	1.401 ± 0.013
Negative Control	DMEM culture medium	1.382 ± 0.028

Proliferation index (PI %) was assessed following treatment. NC (negative control); PC (positive control). Data represent mean ± SEM from three independent experiments. Statistical significance compared to the untreated control was determined by one-way ANOVA followed by Dunnett’s post hoc test.

## Data Availability

The original data presented in the study are included in the article; further inquiries can be directed at the corresponding author.

## Supplementary Materials

The following supporting information can be downloaded at https://www.mdpi.com/article/10.3390/jox16030098/s1. Table S1: Slope assay results for Lambda-Cyhalothrin 5% EC and Duer SC formulations. Cell viability pro-portions were logit-transformed and regressed against log_10_ concentration to estimate the concen-tration-response slope for each formulation; Table S2: One-way ANOVA with Dunnett’s post-hoc test results for cytotoxicity (Figure 1). Mean viability at each concentration was compared to the negative control (untreated cells) for each formulation separately; Table S3: One-way ANOVA with Dunnett’s post-hoc test results for comet assay parameters (Figure 3). Mean values of each DNA damage parameter at each concentration were compared to the nega-tive control (PBS) for each formulation separately.

## References

[1] Damalas C.A., Eleftherohorinos I.G. (2011). Pesticide Exposure, Safety Issues, and Risk Assessment Indicators. Int. J. Environ. Res. Public Health.

[2] Pedroso T.M.A., Benvindo-Souza M., de Araújo Nascimento F., Woch J., dos Reis F.G., de Melo e Silva D. (2022). Cancer and Occupational Exposure to Pesticides: A Bibliometric Study of the Past 10 Years. Environ. Sci. Pollut. Res..

[3] Kaur K., Kaur R. (2018). Occupational Pesticide Exposure, Impaired DNA Repair, and Diseases. Indian J. Occup. Environ. Med..

[4] World Health Organization (WHO) Hazard Identification and Characterization: Toxicology and Human Studies, of Environmental Health Criteria 240 (EHC 240). https://www.who.int/docs/default-source/food-safety/publications/section4-5-genotoxicity.pdf.

[5] Grillo R., Pereira A.E.S., Nishisaka C.S., De Lima R., Oehlke K., Greiner R., Fraceto L.F. (2014). Chitosan/Tripolyphosphate Nanoparticles Loaded with Paraquat Herbicide: An Environmentally Safer Alternative for Weed Control. J. Hazard. Mater..

[6] Nagy K., Tessema R.A., Budnik L.T., Ádám B. (2019). Comparative Cyto- and Genotoxicity Assessment of Glyphosate and Glyphosate-Based Herbicides in Human Peripheral White Blood Cells. Environ. Res..

[7] Nagy K., Rácz G., Matsumoto T., Ádány R., Ádám B. (2014). Evaluation of the Genotoxicity of the Pyrethroid Insecticide Phenothrin. Mutat. Res. Genet. Toxicol. Environ. Mutagen..

[8] Alleva R., Manzella N., Gaetani S., Bacchetti T., Bracci M., Ciarapica V., Monaco F., Borghi B., Amati M., Ferretti G. (2018). Mechanism Underlying the Effect of Long-Term Exposure to Low Dose of Pesticides on DNA Integrity. Environ. Toxicol..

[9] Fetoui H., Feki A., Salah G.B., Kamoun H., Fakhfakh F., Gdoura R. (2015). Exposure to Lambda-Cyhalothrin, a Synthetic Pyrethroid, Increases Reactive Oxygen Species Production and Induces Genotoxicity in Rat Peripheral Blood. Toxicol. Ind. Health.

[10] Fait A., Iversen B., Tiramani M., Visentin S., Maroni M. (2001). Preventing Health Risks form the Use of Pesticides in Agriculture.

[11] Bastos P.L., de Lima Bastos A.F.T., Gurgel A.D.M., Gurgel I.G.D. (2020). Carcinogenicity and Mutagenicity of Malathion and Its Two Analogues: A Systematic Review. Cienc. Saude Coletiva.

[12] Feulefack J., Khan A., Forastiere F., Sergi C.M. (2021). Parental Pesticide Exposure and Childhood Brain Cancer: A Systematic Review and Meta-Analysis Confirming the IARC/WHO Monographs on Some Organophosphate Insecticides and Herbicides. Children.

[13] Nagy K., Duca R.C., Lovas S., Creta M., Scheepers P.T.J., Godderis L., Ádám B. (2020). Systematic Review of Comparative Studies Assessing the Toxicity of Pesticide Active Ingredients and Their Product Formulations. Environ. Res..

[14] Jorgenson B.C., Young T.M. (2010). Formulation Effects and the Off-Target Transport of Pyrethroid Insecticides from Urban Hard Surfaces. Environ. Sci. Technol..

[15] Li X., Liu Y., He L., Gao Y., Mu W., Zhang P., Li B., Liu F. (2020). Fungicide Formulations Influence Their Control Efficacy by Mediating Physicochemical Properties of Spray Dilutions and Their Interaction with Target Leaves. J. Agric. Food Chem..

[16] Kentucky Pesticide Safety Education Program Pesticide Formulations (General Fact Sheet). https://publications.mgcafe.uky.edu/files/ENT70.pdf.

[17] National Pesticide Information Center (NPIC) Lambda-Cyhalothrin (General Fact Sheet). http://npic.orst.edu/factsheets/l_cyhalogen.pdf.

[18] Opinion Regarding the Evaluation of Lambda-Cyhalothrin in the Context of Council Directive 91/414/EEC Concerning the Placing of Plant Protection Products on the Market (Opinion Expressed by the Scientific Committee on Plants, 28 January 2000) Terms of Reference. https://ec.europa.eu/food/plant/pesticides/eu-pesticides-database/backend/api/active_substance/download/478.

[19] Saleh M., Ezz-Din D., Al-Masri A. (2021). In Vitro Genotoxicity Study of the Lambda-Cyhalothrin Insecticide on Sf9 Insect Cells Line Using Comet Assay. Jordan J. Biol. Sci..

[20] Al-Amoudi W.M. (2018). Toxic Effects of Lambda-Cyhalothrin, on the Rat Thyroid: Involvement of Oxidative Stress and Ameliorative Effect of Ginger Extract. Toxicol. Rep..

[21] World Health Organization-IPCS Environmental Health Criteria 99: Cyhalothrin. https://iris.who.int/server/api/core/bitstreams/59208e19-f04d-4db9-a9d9-0047561c7649/content.

[22] Drew W., Harper K., Parmar A., Dow M. Lambda-Cyhalothrin. Human Health Risk Assessment for the Proposed Food/Feed Uses of the Insecticide on Cucurbit Vegetables (Group 9), Tuberous and Corm Vegetables (Subgroup 1C), Grass Forage, Fodder, and Hay (Group 17), Barley, Buckwheat, Oat, Rye, Wild Rice, and Pistachios. Petition Numbers 5F6994, 3E6593, and 6E7077. https://www3.epa.gov/pesticides/chem_search/cleared_reviews/csr_PC-128897_18-Jul-07_a.pdf.

[23] Rizzati V., Briand O., Guillou H., Gamet-Payrastre L. (2016). Effects of Pesticide Mixtures in Human and Animal Models: An Update of the Recent Literature. Chem. Biol. Interact..

[24] Cox C., Surgan M. (2006). Unidentified Inert Ingredients in Pesticides: Implications for Human and Environmental Health. Environ. Health Perspect..

[25] Organization for Economic Cooperation and Development (OECD) Test No. 487: In Vitro Mammalian Cell Micronucleus Test. https://www.oecd.org/en/publications/2023/07/test-no-487-in-vitro-mammalian-cell-micronucleus-test_g1g6fb2a.html.

[26] Organisation for Economic Co-Operation and Development OECD Test No. 489 Guideline for Testing of Chemicals: In Vivo Mammalian Alkaline Comet Assay. http://www.oecd.org/termsandconditions/.

[27] Organisation for Economic Co-Operation and Development (OECD) OECD Existing Chemicals Database Cooperative Chemicals Assessment Programme. https://hpvchemicals.oecd.org/ui/Default.aspx.

[28] Fenech M. (2007). Cytokinesis-Block Micronucleus Cytome Assay. Nat. Protoc..

[29] Fenech M., Kirsch-Volders M., Natarajan A.T., Surralles J., Crott J.W., Parry J., Norppa H., Eastmond D.A., Tucker J.D., Thomas P. (2011). Molecular Mechanisms of Micronucleus, Nucleoplasmic Bridge and Nuclear Bud Formation in Mammalian and Human Cells. Mutagenesis.

[30] Collins A.R. (2004). The Comet Assay for DNA Damage and Repair. Mol. Biotechnol..

[31] Azqueta A., Collins A.R. (2013). The Essential Comet Assay: A Comprehensive Guide to Measuring DNA Damage and Repair. Arch. Toxicol..

[32] Varghese D.S., Oommen D., John A., Ali B.R. (2023). GRP78/BiP Alleviates OxLDL-Induced Hepatotoxicity in Familial Hypercholesterolemia Caused by Missense Variants of LDLR in a HepG2 Cellular Model. Lipids Health Dis..

[33] Naravaneni R., Jamil K. (2005). Evaluation of Cytogenetic Effects of Lambda-Cyhalothrin on Human Lymphocytes. J. Biochem. Mol. Toxicol..

[34] Ilboudo S., Fouche E., Rizzati V., Toé A.M., Gamet-Payrastre L., Guissou P.I. (2014). In Vitro Impact of Five Pesticides Alone or in Combination on Human Intestinal Cell Line Caco-2. Toxicol. Rep..

[35] Bolognesi C., Fenech M., Dhawan A., Bajpayee M. (2019). Micronucleus Cytome Assays in Human Lymphocytes and Buccal Cells. Genotoxicity Assessment: Methods and Protocols.

[36] Xu X., Yu Y., Ling M., Ares I., Martínez M., Lopez-Torres B., Maximiliano J.E., Martínez-Larrañaga M.R., Wang X., Anadón A. (2023). Oxidative Stress and Mitochondrial Damage in Lambda-Cyhalothrin Toxicity: A Comprehensive Review of Antioxidant Mechanisms. Environ. Pollut..

[37] Laborde M.R.R., Larramendy M.L., Soloneski S. (2023). Cytotoxic and Genotoxic Profiles of the Pyrethroid Insecticide Lambda-Cyhalothrin and Its Microformulation Karate® in CHO-K1 Cells. Mutat. Res. Genet. Toxicol. Environ. Mutagen..

[38] Muranli F.D.G. (2013). Genotoxic and Cytotoxic Evaluation of Pyrethroid Insecticides λ-Cyhalothrin and α-Cypermethrin on Human Blood Lymphocyte Culture. Bull. Environ. Contam. Toxicol..

[39] Mauricio-Gutiérrez A., Ramírez-Gutiérrez D.D., Romero-Arenas O., Contreras-Paredes C.A., Mora-Ravelo S., Cedillo-Ramírez L., Yáñez-Santos J.A., Valencia de Ita M.A. (2025). Cytotoxic Effects and Micronuclei Frequency as a Biomarker of Genotoxicity in Farmers from the Municipality of Tehuacán, Puebla, Mexico. Toxics.

[40] Moffat C., Buckland S.T., Samson A.J., McArthur R., Chamosa Pino V., Bollan K.A., Huang J.T.J., Connolly C.N. (2016). Neonicotinoids Target Distinct Nicotinic Acetylcholine Receptors and Neurons, Leading to Differential Risks to Bumblebees. Sci. Rep..

[41] Ramadhan Makame K., Sherif M., Östlundh L., Sándor J., Ádám B., Nagy K. (2023). Are Encapsulated Pesticides Less Harmful to Human Health than Their Conventional Alternatives? Protocol for a Systematic Review of in Vitro and in Vivo Animal Model Studies. Environ. Int..

[42] Li W., Naeem M., Cui J., Du G., Chen H. (2025). Toxicity and Sublethal Effects of Lambda-Cyhalothrin Insecticide on Parent and Filial Generations of *Henosepilachna vigintioctomaculata* (Coleoptera: Coccinellidae). Insects.

[43] Elsharkawy E.E., El-Nasser M.A., Yahia D., Sayed M.M., Bakheet A.A., Abdel-Ghaffar S.K. (2025). DNA Damage and Oxidative Stress Induced by Lambda-Cyhalothrin versus Nano Lambda-Cyhalothrin in Rats. Health Nanotechnol..

[44] Londoño-Velasco E., Asencio-Santofimio H., Ortega-Avila J.G., Rosero-Caldón A.B., Aristizabal-Grisales J.C., Rey-Henao L., Vargas-Rivera J.A., Vergara-Escudero E. (2024). Assessment of Buccal Mucosa Genotoxicity in Insecticide-Exposed Urban Fumigators in Cali, Colombia. Int. J. Occup. Med. Environ. Health.

[45] IARC-WHO IARC Monograph on Glyphosate. https://www.iarc.who.int/featured-news/media-centre-iarc-news-glyphosate/.

[46] Huang X., Wang A., Luo J., Gao Y., Guan L., Zhang P., Liu F., Mu W., Li B. (2022). Lambda-Cyhalothrin-Loaded Nanocapsules Pose an Unacceptable Acute Toxicological Risk to Zebrafish (*Danio rerio*) at the Adult and Larval Stages but Present an Acceptable Risk to Embryos. J. Hazard. Mater..

[47] Bossou Y.M., Côté J., Morin É., Dumais É., Bianchi C., Bouchard M. (2023). Assessing the Impact of Coexposure on the Measurement of Biomarkers of Exposure to the Pyrethroid Lambda-Cyhalothrin in Agricultural Workers. Int. J. Hyg. Environ. Health.

[48] Hou R., Zhou J., Song Z., Zhang N., Huang S., Kaziem A.E., Zhao C., Zhang Z. (2023). PH-Responsive λ-Cyhalothrin Nanopesticides for Effective Pest Control and Reduced Toxicity to *Harmonia axyridis*. Carbohydr. Polym..

[49] Ganilho C., da Silva M.B., Paiva C., de Menezes T.I., dos Santos M.R., Pereira C.M., Pereira R., Andreani T. (2022). Environmental Safety Assessments of Lipid Nanoparticles Loaded with Lambda-Cyhalothrin. Nanomaterials.

