# Supplementary material for: Formulation Matters: Differential Genotoxic and Cytotoxic Effects of Lambda-Cyhalothrin Pesticide Formulations on Human Hepatocellular Cells
Source: J Xenobiot. 2026 May 31;16(3):98. doi: 10.3390/jox16030098 (PMC13301285; doi:10.3390/jox16030098)
Supplement: Supplementary file 1 [file jox-16-00098-s001.zip › jox-4290977-supplementary materials.pdf]

Supplementary Materials: Formulation Matters: Differential Genotoxic and Cytotoxic Effects of Lambda-Cyhalothrin Pesticide Formulations on Human Hepatocellular Cells

Khadija Ramadhan Makame, Moustafa Sherif, Le Vinh Hoi Thong, Balázs Ádám and Károly Nagy

Supplementary Table S1. Slope assay results for Lambda-Cyhalothrin 5% EC and Duer SC formulations. Cell viability proportions were logit-transformed and regressed against $\log_{10}$ concentration to estimate the concentration-response slope for each formulation.

Formulation	Slope (logit-viability per $\log_{10}$ µM)	Standard Error	P_value	Significant Negative Slope
Lambda-Cyhalothrin 5% EC	-0.3666	0.0671	8.32E-05	YES, significant toxicity
Duer SC	-0.3395	0.0458	3.31E-06	YES, significant toxicity

Slope estimated from linear regression of logit-transformed viability [$l = \ln((p + 0.005)/(1 - p + 0.005))$] on $\log_{10}$ concentration. A significant negative slope indicates concentration-dependent reduction in cell viability. Statistical significance threshold: $p < 0.05$.

Supplementary Table S2. One-way ANOVA with Dunnett's post-hoc test results for cytotoxicity (Figure 1). Mean viability at each concentration was compared to the negative control (untreated cells) for each formulation separately.

Formulation	Concentration (µM)	Mean Difference (%)	Standard Error	P_value	Significance
Lambda-Cyhalothrin 5% EC	0.01	0.695	3.648	1	ns
	0.1	1.294	3.648	1	ns
	1	0.249	3.648	1	ns
	10	-6.493	3.648	0.46	ns
	100	-8.291	3.648	0.232	ns
	200	-9.756	3.648	0.123	ns
	500	-20.722	3.648	0.00105	**
	1000	-29.763	3.648	4.00E-05	***
Duer SC	0.01	2.288	2.767	0.964	ns
	0.1	2.677	2.767	0.923	ns
	1	-1.96	2.767	0.984	ns
	10	-4.18	2.767	0.625	ns
	100	-8.706	2.767	0.0568	ns
	200	-10.474	2.767	0.0195	*
	500	-18.042	2.767	0.000268	***
	1000	-22.181	2.767	3.44E-05	***

Mean difference = treated − negative control. Negative values indicate reduction in viability relative to untreated cells. Significance: *$p < 0.05$, **$p < 0.01$, ***$p < 0.001$; ns = not significant. Analysis performed using one-way ANOVA followed by Dunnett's post-hoc test with the untreated negative control as reference group.

https://doi.org/10.3390/jox16030098

Supplementary Table S3. One-way ANOVA with Dunnett's post-hoc test results for comet assay parameters (Figure 4). Mean values of each DNA damage parameter at each concentration were compared to the negative control (PBS) for each formulation separately.

Formulation	Parameter	Concentration (µM)	Mean Difference	Standard Error	P_value	Significance
Lambda-Cyhalothrin 5% EC	% DNA in Tail	0.01	-0.2946	5.0069	1	ns
		0.1	3.1637	5.0069	0.993	ns
		1	4.8919	5.0069	0.927	ns
		10	18.5341	5.0069	0.00889	**
		50	26.0992	5.0069	0.000245	***
		100	53.5772	5.0069	1.78E-10	***
		200	55.8932	5.0069	4.27E-11	***
	Tail Length (µm)	0.01	-5.3734	8.7832	0.994	ns
		0.1	-1.7078	8.7832	1	ns
		1	8.901	8.7832	0.913	ns
		10	28.9757	8.7832	0.0226	*
		50	46.7216	8.7832	0.00019	***
		100	63.2816	8.7832	2.59E-06	***
		200	53.0394	8.7832	3.28E-05	***
	Tail Moment	0.01	-1.7323	4.479	1	ns
		0.1	-0.7451	4.479	1	ns
		1	1.5057	4.479	1	ns
		10	16.553	4.479	0.00909	**
		50	32.2022	4.479	1.69E-06	***
		100	72.384	4.479	0	***
		200	62.6543	4.479	1.54E-13	***
	Olive Tail Moment	0.01	-0.6704	1.4993	0.999	ns
		0.1	-0.1613	1.4993	1	ns
		1	0.389	1.4993	1	ns
		10	5.0992	1.4993	0.0179	*
		50	10.7582	1.4993	2.23E-06	***
		100	26.4828	1.4993	0	***
		200	24.328	1.4993	1.03E-13	***
Duer SC	% DNA in Tail	0.01	2.8646	5.2104	0.997	ns
		0.1	2.9623	5.2104	0.996	ns
		1	1.3928	5.2104	1	ns
		10	5.7219	5.2104	0.877	ns
		50	4.8168	5.2104	0.944	ns
		100	-2.4406	5.2104	0.999	ns
		200	13.202	5.2104	0.118	ns
	Tail Length (µm)	0.01	-0.069	9.6845	1	ns
		0.1	-1.0609	9.6845	1	ns
		1	-4.2953	9.6845	0.999	ns
		10	2.7125	9.6845	1	ns
		50	12.006	9.6845	0.802	ns
		100	-11.799	9.6845	0.815	ns
		200	29.4716	9.6845	0.0401	*
	Tail Moment	0.01	-0.5536	4.9002	1	ns
		0.1	-0.9772	4.9002	1	ns

https://doi.org/10.3390/jox16030098

		1	-0.884	4.9002	1	ns
		10	0.9486	4.9002	1	ns
		50	1.566	4.9002	1	ns
		100	-3.5283	4.9002	0.985	ns
		200	13.5402	4.9002	0.0737	ns
	Olive Tail Moment	0.01	-0.2041	1.517	1	ns
		0.1	-0.2256	1.517	1	ns
		1	-0.4063	1.517	1	ns
		10	0.3694	1.517	1	ns
		50	-0.0334	1.517	1	ns
		100	-1.2706	1.517	0.965	ns
		200	3.8207	1.517	0.122	ns

Mean difference = treated − negative control. Positive values indicate increased DNA damage relative to control. Significance: *$p < 0.05$, **$p < 0.01$, ***$p < 0.001$; ns = not significant. Analysis performed using one-way ANOVA followed by Dunnett's post-hoc test with the untreated negative control (PBS) as reference group. For tail length, tail moment, and olive tail moment parameters, variance increased at higher concentrations (consistent with the distributional properties of comet assay data); significance levels for these parameters should therefore be regarded as approximate. PC = hydrogen peroxide (H_2O_2, 100 µM).

GLM MODEL – BINARY LOGISTIC REGRESSION

```
> summary(logit_model_log)

Call:
glm(formula = Lambda_binary ~ logConc, family = binomial, data = df)

Coefficients:
             Estimate Std. Error z value Pr(>|z|)
(Intercept)     68.32  120133.72   0.001        1
logConc        -45.52   75173.10  -0.001        1

(Dispersion parameter for binomial family taken to be 1)

    Null deviance: 9.5607e+00  on 6  degrees of freedom
Residual deviance: 5.2147e-10  on 5  degrees of freedom
AIC: 4

Number of Fisher Scoring iterations: 24
```

GLM model yields very high inflate standard errors due to the small sample size
